# Cyclic Stretch Enhances Osteogenic Differentiation of Human Periodontal Ligament Cells via YAP Activation

**DOI:** 10.1155/2018/2174824

**Published:** 2018-11-05

**Authors:** Yang Yang, Bei-Ke Wang, Mao-Lin Chang, Zi-Qiu Wan, Guang-Li Han

**Affiliations:** ^1^State Key Laboratory Breeding Base of Basic Science of Stomatology (Hubei-MOST) and Key Laboratory for Oral Biomedicine of Ministry of Education (KLOBM), School and Hospital of Stomatology, Wuhan University, 237 Luoyu Road, Wuhan 430079, China; ^2^Department of Orthodontics, School & Hospital of Stomatology, Wuhan University, Wuhan, China

## Abstract

Periodontal remodeling and alveolar bone resorption and formation play essential roles during orthodontic tooth movement (OTM). In the process, human periodontal ligament cells (HPDLCs) sense and respond to orthodontic forces, contributing to the alveolar bone formation. However, the underlying mechanism in this process is not fully elucidated. In the present study, cyclic stress stimulus was applied on HPDLCs to mimic the orthodontic forces during OTM. Our results demonstrated that cyclic stretch promoted the osteogenic differentiation of HPDLCs. Moreover, our data suggested that yes-associated protein (YAP), the Hippo pathway effector, which also involved in mechanical signaling transduction, was activated as we found that the nuclear translocation of YAP was significantly increased in the cyclic stress treated HPDLCs. The mRNA expression of CTGF and CYR61, the target genes of YAP, was also remarkably increased. Furthermore, knockdown of YAP suppressed the cyclic stretch induced osteogenesis in HPDLCs, while overexpression of YAP in HPDLCs enhanced osteogenesis. We also noticed that YAP activities could be suppressed by the ROCK and nonmuscle myosin II inhibitors, Y-27632 and Blebbistatin. The inhibitors also significantly inhibited the cyclic stretch induced osteogenesis in HPDLCs. Finally, in the murine OTM model, our results revealed that YAP was upregulated and nuclearly translocated in the PDLCs at the tension side. In summary, our present study demonstrated that cytoskeleton remodeling induced activation of YAP signaling pathway was crucial for the cyclic stretch-induced osteogenesis of HPDLCs, which might play important roles during OTM.

## 1. Introduction

Extracellular mechanical stimuli, including extracellular matrix stiffness, stretch, or shear stress, can be sensed by the cells, which further regulate cell proliferation and differentiation and may contribute to tumor progression [1, 2]. During the process of orthodontic tooth movement (OTM), periodontal ligament (PDL), the connective tissue localized between tooth cementum and alveolar bone, sensed the orthodontic force and mediated the bone formation at the tension side while the bone resorption at the compressive side [3–5]. It has been reported that the periodontal ligament cells (PDLCs) were able to sense the mechanical signals and mediate the remodeling of periodontal ligament and alveolar bone. Besides, it is also believed that PDLCs contribute to the new bone formation at the tension side via transdifferentiation into the osteoblasts [6]. However, the underlying mechanism by which PDLCs differentiate into osteoblasts during OTM is largely unknown.

Several signaling pathways, including FAK/MAPK and Rho/ROCK signaling pathways, are involved in the mechanical signaling transduction [7]. Recently, yes-associated protein (YAP) and the paralogue transcriptional coactivator with PDZ-binding motif (TAZ), the downstream effectors of the Hippo signaling pathway, have been identified as the crucial regulators during mechanotransduction [1]. YAP senses the extracellular mechanical cues, including the ECM stiffness, stretch and stress forces, and translocates into nucleus, acting as the coactivator of many other transcription factors to regulate the downstream gene expression and reprogram the cells. Otherwise, the cytoplasmic YAP is generally degraded under the control of Hippo signaling pathways [8].

Emerging studies have reported that YAP was involved in the regulation of cell proliferation, organ size control, cell differentiation and oncogenesis [9–11]. As a coactivator, YAP is able to interact with TEAD domain family member, p73, Runt-related transcription factor 2 (RUNX2), T-box 5 (TBX5) and facilitates the transcription of their downstream genes [12–14]. By virtue of the coactivator function, YAP is involved in the regulation of osteoblastic differentiation of mesenchymal stem cells (MSCs). Chan LH et al. reported that YAP overexpression promoted the osteogenesis by upregulating the expression of RUNX2 and Osteocalcin in a mouse model [15]. In addition, Zhang Y et al. reported that the depletion of YAP was found to decrease the grid topology (GT) substrates-induced osteoblastic differentiation of MC3T3-E1 cells by attenuating alkaline phosphatase (ALP) activity [16]. It has also been reported that TAZ, the paralogue of YAP, also promoted the osteoblastic differentiation by stimulating RUNX2-mediated gene transcription [17]. Therefore, we proposed that the orthodontic mechanical stimulus during the OTM might activate YAP, and further promote osteogenic differentiation of PDLCs.

In the present study, we reported that YAP was activated in the PDLCs which were treated with cyclic stretch force, mimicking the orthodontic force during the OTM at the tension side. Moreover, out data suggested that activation of YAP was dependent on the cytoskeleton remodeling and the upregulation of YAP was efficient to induce the osteogenic differentiation of PDLCs. Depletion of YAP inhibited the cyclic stress-induced osteogenesis in PDLCs. Furthermore, the increased expression of YAP was observed in the experimental OTM mice model. This study may elucidate the mechanisms involved in the process of osteoblastic differentiation regulated by tensile force and provide further insights in the improvement of orthodontic treatment.

## 2. Materials and Methods

### 2.1. HPDLCs Isolation, Culture, and Induction of Osteoblastic Differentiation

Human periodontal ligament cells (HPDLCs) were isolated and cultured according to the previously reported protocol [18, 19]. Briefly, disease-free premolars (n=10) were extracted for orthodontic purpose from both male and female healthy volunteers aged 12-24 years in the Hospital of Stomatology, Wuhan University. The basic information of the healthy donors has been shown in Supplementary Table  1. Periodontal ligament tissues were gently scraped off from the root surface of the tooth and cut into smaller pieces. Then, the treated tissues were digested in a mixed solution containing 6 mg/ml collagenase type I (Sigma-Aldrich, St. Louis, MO) and 8 mg/ml dispase (Sigma-Aldrich, St. Louis, MO) for 60 min. Single cell suspension was then harvested by filtering through a 70-*μ*m cell strainer (BD Biosciences, Franklin Lakes, NJ) and centrifuged at 1500 rpm for 5 min to remove the supernatant. The cell pellets were resuspended and then incubated in alpha-modified Eagle's Medium (*α*-MEM, HyClone, Logan, Utah) supplemented with 10% fetal bovine serum (FBS, Hyclone, Logan, Utah) and antibiotics (100 U/ml penicillin and 100 *μ*g/ml streptomycin, HyClone, Logan, Utah) at 37°C in a humidified incubator with 95% air and 5% CO_2_. HPDLCs between passages 3 and 8 were used in this study. All the procedures and protocols were performed in accordance with the NIH guidelines regarding the use of human tissues and approved by the review board of the Ethics Committee of the Hospital of Stomatology, Wuhan University. All the patients participated in the study had been provided informed consent before tooth extraction.

For osteogenic differentiation induction, the culture medium of HPDLCs was replaced with osteogenic differentiation media (*α*-MEM containing 10 mM *β*-glycerophosphate, 0.5 *μ*M dexamethasone, 50 mg/mL ascorbic acid, and 10% FBS) when the cells reached confluent condition. The differentiation medium was refreshed every 2 days [20].

### 2.2. Application of Cyclic Stretch to HPDLCs

To mimic the tensile force exerted on HPDLCs during OTM, a custom-made strain device Tension Plus System, was used [19]. The tension system has been further modified in the present study by positioning six planar faced posts beneath the silicon membranes of the culture wells to provide the uniform strain to the cultured cells. Equal numbers of HPDLCs were seeded into the six-well, flexible-bottomed plate coated with type I collagen (BioFlex, Flexcell International Corp, Hillsborough, NC). After reaching 80% confluence, the medium was refreshed and cyclic tensile force (10% bottom membrane deformation) was applied to HPDLCs at a frequency of 0.1 Hz (5 s stress and 5 s rest) for 24 h for mRNA extraction and 72 h for ALP staining, western blots analyses and immunofluorescence detections. HPDLCs that were maintained in identical conditions without being stretched were used as control. After stretch application procedure, cells were harvested for protein or mRNA expression analyses or were subjected to ALP staining assays.

### 2.3. RNA Extraction, cDNA Synthesis, and Quantitative Real-Time PCR Analysis

The total RNA of HPDLCs was isolated by TRIzol reagent (TR118, Molecular Research Center, Cincinnati, Ohio) and the first-strand cDNA was synthesized using a RevertAid First Strand cDNA Synthesis Kit (K1622, Thermo Fisher Scientific, Waltham, MA). Polymerase chain reactions (PCR) were performed using FastStart Universal SYBR Green Master (ROX) (Roche, Penzberg, Germany) within QuantStudio 6 Flex Real-Time PCR System (Applied Biosystems, Foster City, CA). The ^ΔΔ^Ct method was adopted to calculate the relative mRNA levels of the genes that were normalized to GAPDH. Primers used in this study were synthesized by TsingKe Biological Technology (Wuhan, China) and were shown as follows: GAPDH, 5′-ACAACTTTGGTATCGTGGAAGG-3′ and 5′-GCCATCACGCCACAGTTTC-3′; OPN, 5′-GAAGTTTCGCAGACCTGACAT-3′ and 5′-GTATGCACCATTCAACTCCTCG-3′; ALP, 5′-ACTGGTACTCAGACAACGAGAT-3′ and 5′-ACGTCAATGTCCCTGATGTTATG-3′; Collagen type 1, 5′-CCCCCTCCCCAGCCACAAAG-3′ and 5′-TCTTGGTCGGTGGGTGACTCT-3′; OSX, 5′-GAGGCAACTGGCTAGGTGG-3′ and 5′-CTGGATTAAGGGGAGCAAAGTC-3′; YAP, 5′-CCTGATGGATGGGAACAAGC-3′ and 5′-GCACTCTGACTGATTCTCTGG-3′; RUNX2, 5′-TGGTTACTGTCATGGCGGGTA-3′ and 5′-TCTCAGATCGTTGAACCTTGCTA-3′; OCN 5′-CACTCCTCGCCCTATTGGC-3′ and 5′-CCCTCCTGCTTGGACACAAAG-3′; CYR61, 5′-ATGAATTGATTGCAGTTGGAAA-3′ and 5′-TAAAGGGTTGTATAGGATGCGA-3′; CTGF, 5′-CCCAAGGACCCAAACCGTG-3′ and 5′-CTAATCATAGTTGGGTCTGGGC-3′.

### 2.4. Immunoblot Analysis

Total protein of HPDLCs was lysed with mammalian protein extraction reagent (M-PER) (Thermo Fisher Scientific, Waltham, MA) that was supplemented with protease inhibitors (Roche, Penzberg, Germany). Nuclear protein of HPDLCs was extracted using a Nuclear and Cytoplasmic Protein Extraction Kit (P0028, Beyotime, China). The concentration of protein was detected by BCA assays. 30 *μ*g of protein with loading buffer was loaded and separated on 12% sodium dodecyl sulfate-polyacrylamide gel electrophoresis (SDS-PAGE) gels and transferred onto polyvinyldifluoride (PVDF) membranes (Millipore, Bedford, MA). The membranes were blocked with 5% skimmed milk at room temperature for 1 h and incubated with appropriate primary antibodies at 4°C overnight. The membranes were then incubated with secondary antibody conjugated to horseradish peroxidase (HRP) (PMK-013-09C, PM, China, 1:10000) at room temperature for 1 h. Immunoreactivity was visualized using western blotting detection kit (Advansta, Menlo Park, CA). The integrated optical density (IOD) values of each band were analyzed using ImageJ software (National Institutes of Health, Bethesda, MD). The band intensity was normalized to that of GAPDH and expressed as a relative ratio. The primary antibodies used in the present study were as follows: anti-YAP (ABclonal, A1002, 1:2000), anti-OCN (ABclonal, A1530, 1:4000), anti-OPN (Abclonal, A1361, 1:4000), and GAPDH (PMK053F, PM, China, 1:3000).

### 2.5. Immunofluorescence Analysis

After culturing and being stretched in the flexible-bottomed culture plates, HPDLCs were fixed in 4% paraformaldehyde for 10 min at room temperature, the cells were then permeabilized with 0.5% Triton X-100 in phosphate-buffered saline (PBS). The permeabilized cells were incubated with a YAP antibody (ABclonal, A1002, 1:200) diluted in PBS at 4°C overnight. Washed with PBS for 10 min for 3 times, the cells were probed with goat anti-rabbit IgG conjugated with Cy3 in darkness for 1 h. Finally, nuclei were counterstained with 6-diamidino-2-phenylindole (DAPI). The fluorescent images were acquired using a Leica DM4000B fluorescence microscope (Leica, Nussloch, Germany) and analyzed by ImageJ (https://imagej.nih.gov/ij/).

### 2.6. RNA Interference

For small interfering RNA (siRNA) transfection, siRNAs were designed and synthesized in RNA oligo duplex with dTdT modification on both 5′ and 3′ ends (Genepharma, Shanghai, China). Sense strand sequences are designed as follows: YAP siRNA #1, 5′-GACGACCAAUAGCUCAGAU-3′; YAP siRNA #2, 5′-GACAUCUUCUGGUCAGAGA-3′; negative control sense, 5′-UUCUCCGAACGUGUCACGU-3′. The siRNA duplexes (30 nM) were transfected into HPDLCs using Pepmute siRNA transfection reagent (SignaGen, MD) according to the manufacturer's instructions. The expression levels of mRNA and protein were analyzed 48 h after transfection.

### 2.7. Lentiviral Transduction

To generate HPDLCs overexpressing YAP, the YAP cDNA was cloned into the lentiviral expression plasmid pCDH (pCDH-YAP). To produce lentiviruses, 293E packaging cells were transfected with vector or pCDH-YAP plasmids, combined with the packaging plasmids psPAX2 and pMD2.G (Invitrogen, Waltham, MA). The day before transfection, 293E cells were plated in the 10 cm dish cultured in DMEM (Invitrogen, Waltham, MA) with 10% FBS (Hyclone, Logan, Utah). When cell density reached 80%, the cells were transfected with the above plasmids using polyethylenimine (PEI, Polysciences, PA). After the transfected cells were incubated in 37°C, 5% CO_2_ incubator for 48 hours, the culture medium was replaced with 5 ml fresh DMEM with 10% FBS. The supernatants containing viruses were harvested at 48 hours and were stored at −80°C until use.

### 2.8. Alkaline Phosphatase Staining and Activity Assay

HPDLCs were fixed with 4% paraformaldehyde for 10 min at room temperature and washed with PBS for 3 times. To visualize alkaline phosphatase (ALP) staining in the HPDLCs, a BCIP/NBT Alkaline Phosphatase Color Development Kit (C3206, Beyotime, China) was used according to the manufacturer's instruction. BCIP (5-Bromo-4-Chloro-3-Indolyl Phosphate), the common substrate of ALP, could be hydrolyzed by ALP into a product with strong reactivity, which were then reacted with NBT (Nitrotetrazolium Blue chloride) and formed insoluble dark blue or blue-violet NBT-formazan, reflecting the activity of ALP in cells. Images were taken using a digital camera. To quantify the ALP activities of HPDLCs, the cells were washed with PBS and lysed with M-PER. The total protein concentration (mg/ml) and ALP activity levels (U/ml) were measured with an BCA assay kit and an ALP reagent kit (Nanjing Jiancheng Bioengineering Research Institute, Nanjing, China), respectively, according to the manufacturer's instructions. ALP levels were normalized to the total protein content. All samples were performed in triplicate.

### 2.9. Inhibitors

Y27632 (ROCK inhibitor, 20 *μ*M, S1049, Selleck) and Blebbistatin (non-muscle myosin II inhibitor, 20 *μ*M, S7099, Selleck) were used in the present study. Dissolved in DMSO, all inhibitors were added to the culture medium 1 h before application of cyclic stretch.

### 2.10. Experimental Tooth Movement in Mice

The study was approved by the Ethics Committee of the School of Stomatology, Wuhan University, in accordance with local laws and regulations. The experimental tooth movement mouse model was established as we previously reported [19, 21]. Briefly, the animals used in the study were 8-week-old healthy male C57BL/6 mice, which were anesthetized through intraperitoneal injection with 0.4 mg/kg chloral hydrate (Sigma, St. Louis, MO). Experimental tooth movement was performed using a nickel–titanium coil spring (Tomy, Tokyo, Japan), and the distal end of the spring was directly bonded to the occlusal surface of the right first maxillary molar by a light-cured resin (Transbond, 3 M Unitek, St. Paul, MN) while the mesial end was bonded to both upper-incisors. The left side not treated with spring coil was used as control. A continuous force of 0.35 N was applied to the first molar in the mesial direction, and the amount of force generated by the spring was kept by a tension gauge. After 0, 3, 6, and 12 days of experimental tooth movement, animal mice were sacrificed through overdose of anesthetics for immunohistochemistry analysis.

### 2.11. Tissue Processing and Immunohistochemistry Analysis

The maxilla tissue sections were separated and fixed in 4% paraformaldehyde for 24 hours, and then decalcified in 10% EDTA (pH 7.4) for 10 weeks. After demineralization, samples were dehydrated and embedded in paraffin. 5 *μ*m thick mesiodistal serial sections were adopted and mounted onto the polylysine-coated slides. Immunohistochemistry analysis was performed using an UltraSensitive™ SP (Rabbit) IHC Kit (KIT-9706, MXB, China). The sections were deparaffinized, rehydrated, antigen-retrieved by pepsin (DIG-3009, MXB, China), and blocked with 3% goat serum according to the manufacturer's instructions. After that, the samples were incubated with primary antibody diluted with PBS overnight at 4°C. The next day, the tissues were washed with PBS for three times and incubated with biotin-conjugated secondary antibody and streptomyces avidin peroxidase for 20 min, followed by washing for 3 times. Immunoreactivity was visualized using a DAB chromogenic reagent kit (DAB-0031/1031, MXB, China). The images were captured on OLYMPUS BX51 microscopy (OLYMPUS, Japan). The primary antibodies used in the study are as follows: anti-YAP (A1002, ABclonal, 1:1000) and anti-RUNX2 (ab192256, abcam, 1:1200). The biotin-conjugated secondary antibody and avidin peroxidase were included in an IHC detection Kit (KIT-9706, MXB, China) and ready-to-use. For quantification, five tension periodontal tissue areas within each section were selected and analyzed by ImageJ.

### 2.12. Statistical Analysis

All data was expressed as the mean ± standard error (SEM). Statistical significance of the experimental results was performed using unpaired Student's t-tests.* P *< 0.05 was considered statistically significant. All experiments were performed in triplicates.

## 3. Results and Discussion

### 3.1. Cyclic Stretch Induces YAP Nuclear Localization and Activity in HPDLCs

**Figure 1:**
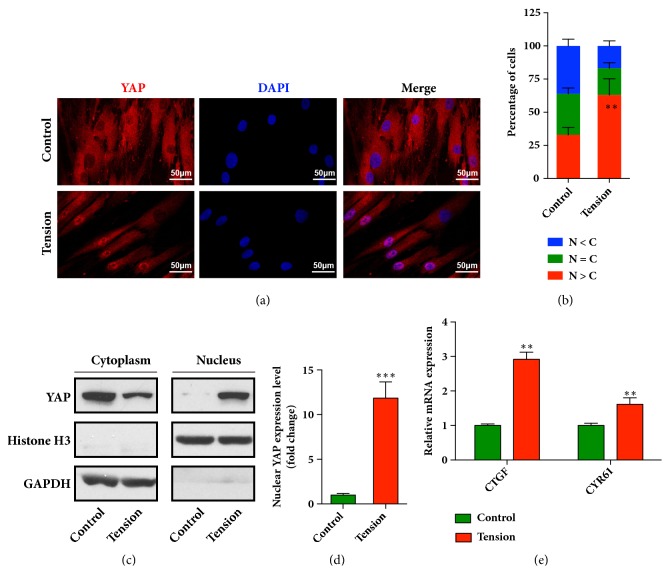
**Cyclic stretch activates YAP in human periodontal ligament cells (HPDLCs)**. (a) Cyclic stretch increased the nuclear localization of YAP in HPDLCs. (b) Quantitative analysis of the cellular distribution of YAP in HPDLCs based on whether it was higher in the nucleus (N>C), higher in the cytoplasm (N<C), or evenly distributed between the nucleus and cytoplasm (N=C). The percentage of cells in each category was scored after observing cells in three different microscopic fields. (c) Nuclear expression of YAP was increased in stretched HPDLCs. (d) Relative grey values of each band (in (c)) were normalized to the level of GAPDH. *∗∗∗*,* p* < 0.001. (e) Increased mRNA expression of YAP downstream genes in stretch-stimulated HPDLCs. The mRNA expression of CTGF and CYR61 was analyzed by qRT-PCR. The relative expression was calculated after normalization to the GAPDH level. *∗∗∗*,* p* < 0.001; *∗∗*,* p *< 0.01. All data was based on three independent experiments.

Cytoskeleton remodeling occurring under the mechanical stimulations from the shear force, stretch force, and others could activate YAP/TAZ signaling pathway and the following various biochemical processes in difference cell types [8]. Recently, YAP/TAZ have been demonstrated as a mechanical regulator to participate in the periodontal tissue remodeling. It was reported that YAP promoted the differentiation and mineralization of cementoblast partly by regulating the Smad-dependent BMP and Erk1/2 signaling pathways [22]. TAZ activated by collagen triple helix repeat containing 1 (CTHRC1) has been reported to modulate the osteoblastic differentiation of periodontal ligament stem cells during orthodontic tooth movement (OTM) [23]. Here, we proposed the idea that, during orthodontic treatment, the activity of YAP and/or TAZ might be increased in the HPDLCs subjected to the orthodontic tensile force. We then explored the nuclear localization of YAP in the stretched HPDLCs using immunofluorescence. As shown in Figure 1(a), the YAP was remarkably concentrated in the nuclei of the cells that were treated by cyclic stress. The enhanced nuclear localization of YAP was found in up to 60% HPDLCs in the tension group, which was around 30% in the control group (Figure 1(b)). By extracting the nuclear protein, we analyzed the nuclear expression of YAP in the stretched HPDLCs using immunoblot assays. The results showed that the nuclear expression of YAP was significantly increased, while the cytoplasmic YAP was slightly decreased (Figures 1(c) and 1(d)). Since YAP acted as a co-transcription factor and thus facilitated the transcription of numerous genes, we next measured the mRNA expression of the downstream genes of TEAD, including CTGF and CYR61, using real time PCR assays. As shown in Figure 1(e), the mRNA expression of CTGF and CYR61 was significantly elevated in the HPDLCs after cyclic stretch treatment by approximately 3 and 1.5 folds, respectively, compared to the control group. In addition, we also examined the activation of TAZ in the cyclic stretched HPDLCs. However, the results from western blots and immunofluorescence revealed the slight accumulation of TAZ in the nuclei of the HPDLCs (Supplementary Fig.  S1), suggesting limited activation of TAZ in the HPDLCs, at least in our cyclic stretching system. Taken together, these results indicated that cyclic stretch promoted the nuclear translocation of YAP and induced its activity in HPDLCs.

### 3.2. Cyclic Stretch Induces Osteoblastic Differentiation of HPDLCs in Our Strain Device Tension Plus System

**Figure 2:**
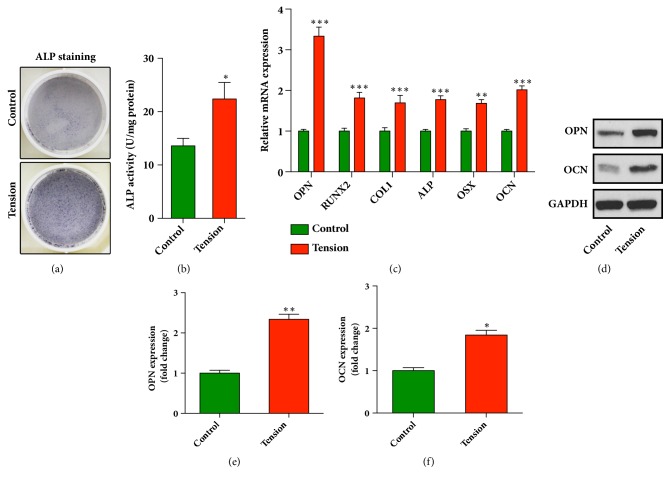
**Cyclic stretch enhances osteoblastic differentiation of HPDLCs**. (a) Cyclic stretch increased the alkaline phosphatase activity of HPDLCs. The positive cells for the ALP were stained with NBT-formazan (dark blue) and the representative image of positive ALP staining is shown in (a). (b) The ALP activity of HPDLCs was quantified using an ALP reagent kit. The activities were normalized to the protein concentration. *∗*,* p* < 0.05. (c) Increased expression of osteogenic marker genes in HPDLCs after cyclic stretch. The mRNA expression of YAP, osteopontin (OPN), RUNX2, collagen type 1 (COL1), alkaline phosphatase (ALP), osterix (OSX), and osteocalcin (OCN) was analyzed using qRT-PCR. The relative expression level was calculated after normalization to the GAPDH level. *∗∗∗*,* p* < 0.001; *∗∗*,* p* < 0.01. (d) The expression of OPN and OCN in HPDLCs was analyzed using immunoblots. (e) Relative grey values of each band in (d) were normalized to the level of GAPDH. *∗∗*,* p* < 0.01; *∗*,* p* < 0.05. All data was based on three independent experiments.

We next explored whether the cyclic stretch, which mimicked the orthodontic force, was able to induce osteogenic differentiation of HPDLCs. To this end, HPDLCs were incubated in osteogenic differentiation media with or without being stretched for 3 days. Then the osteogenic differentiation potential of HPDLCs was measured by alkaline phosphatase (ALP) activity staining and quantitative assays. As shown in Figures 2(a) and 2(b), the alkaline phosphatase activity was significantly increased in the stretched cells but not the control cells. To further investigate the osteogenic potential, the mRNA expression levels of osteogenic marker genes, including osteopontin (OPN), Runt-related transcription factor 2 (RUNX2), collagen type 1, alkaline phosphatase (ALP), osterix (OSX) and osteocalcin (OCN) in the stretched HPDLCs were analyzed using qRT-PCR. As shown in Figure 2(c), the mRNA levels of OPN, RUNX2, collagen type 1, ALP, OSX, and OCN were significantly increased (Figure 2(c)). Moreover, our data from western blots assays showed the remarkable increase of OPN and OCN in the stretched HPDLCs (Figures 2(d) and 2(e)). In summary, these results demonstrated that cyclic stretch significantly promoted the osteoblastic differentiation of HPDLCs.

### 3.3. Depletion of YAP Attenuates Cyclic Stretch-Induced Osteogenic Differentiation of HPDLCs

**Figure 3:**
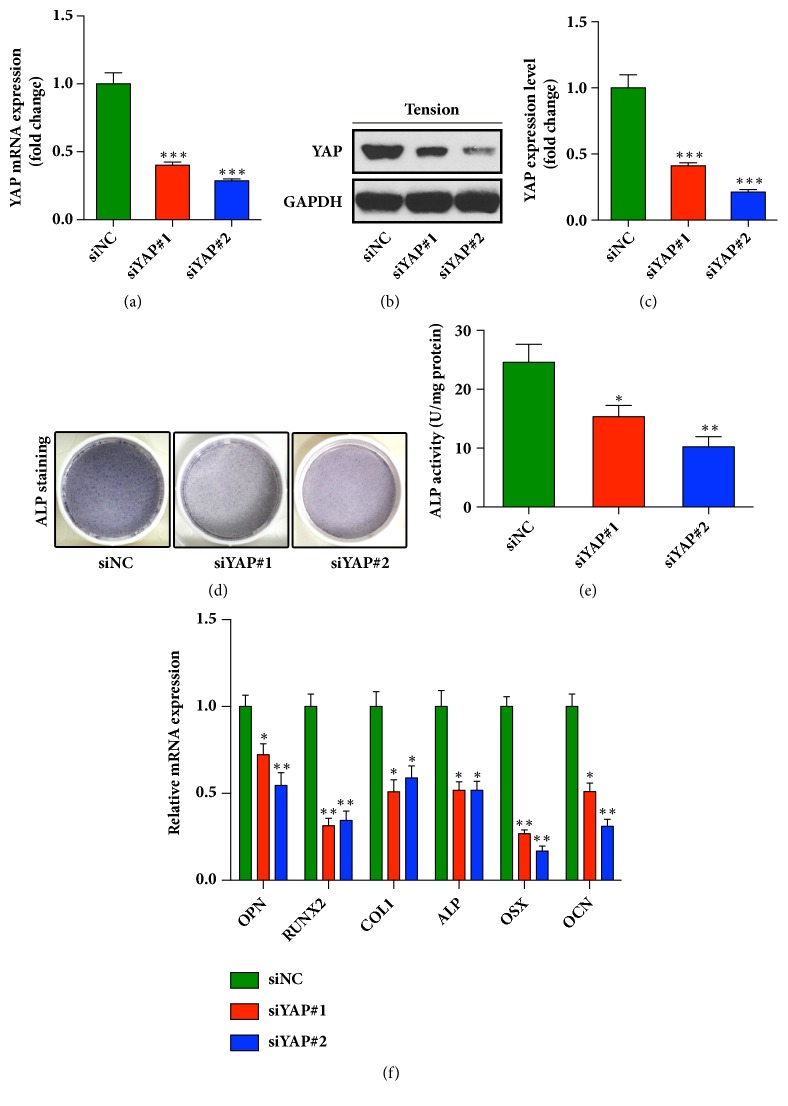
**Cyclic stretch-induced osteogenesis was weakened in the YAP-depleted HPDLCs**. (a) Two different siRNA were used to deplete YAP in HPDLCs. The knockdown efficiency was verified by qRT-PCR. *∗∗∗*, p < 0.001. (b) Depletion of YAP was verified using immunoblot. (c) Quantitative analysis of the blots (in (b)), the data was normalized to the level of GAPDH. *∗∗∗*,* p* < 0.001. (d) YAP knockdown decreased the alkaline phosphatase activity of HPDLCs treated with cyclic stretch. (e) The ALP activity of HPDLCs was measured using an ALP reagent kit. The activities were normalized to the protein concentration. *∗*, P < 0.05; *∗∗*, P < 0.01. (f) mRNA expression of osteogenesis related genes in YAP-depleted HPDLCs. The relative expression levels of osteopontin (OPN), RUNX2, collagen type 1 (COL1), alkaline phosphatase (ALP), osterix (OSX), and osteocalcin (OCN) were normalized to GAPDH. *∗*,* p* < 0.05; *∗∗*,* p* < 0.01. All data was based on three independent experiments.

Previous studies reported that activation of YAP could induce osteogenic differentiation in MSCs [1, 24]. Xu et al. proved that YAP/TAZ was involved in the process of periodontal tissue remodeling during OTM in rat [25]. Furthermore, they demonstrated that knockdown of YAP induced the apoptosis and inhibited the proliferation of human periodontal ligament stem cells (h-PDLSCs) through crosstalk between Erk and Bcl-2 signaling pathway, suggesting that YAP signaling pathway participated in regulating biological behaviors of h-PDLSCs [26]. Diana Huelter-Hassler also found that the activated YAP might contribute to the enhanced proliferation of HPDLCs during orthodontic tooth movement [27, 28]. Importantly, it was reported that the mechanical stress increased the expression levels of osteogenesis-related genes ALP and type I collagen in HPDLCs, and the mechanical stimulation also influenced the maintenance of periodontal ligament and alveolar bone formation [3]. Therefore, we proposed that activation of YAP in HPDLCs might contribute to enhanced osteogenic differentiation of HPDLCs under mechanical stimulation. Two small interfering RNA (siRNA) were used to deplete the YAP expression in HPDLCs. No cytotoxic effect of siRNA has been confirmed via trypan blue exclusion assays (Supplementary Fig.  S2). As shown in Figures 3(a)–3(c), YAP knockdown (KD) significantly decreased mRNA and protein expression of YAP in HPDLCs. Both YAP-depleted cells and control cells were incubated in osteogenic differentiation media and stretched with different time for different detections as described above. After 3 days, the osteoblastic differentiation potential was assessed by ALP activity assays. As shown in Figures 3(d) and 3(e), the osteogenic potential was significantly decreased in the two stretched YAP-KD cells, as the staining for ALP activity was dramatically weakened. In addition, after 1-day tension exposure, the decreased expression of osteogenesis-related marker genes, including OPN, RUNX2, collagen type 1, ALP, OSX, and OCN, was also observed in the YAP knockdown HPDLCs (Figure 3(f)). These results indicated that the stretch induced activation of YAP was important for the osteogenic differentiation potential of HPDLCs and knockdown of YAP could significantly impair the osteogenesis of HPLDCs.

### 3.4. Overexpression of YAP Enhances Osteoblastic Differentiation of HPDLCs

**Figure 4:**
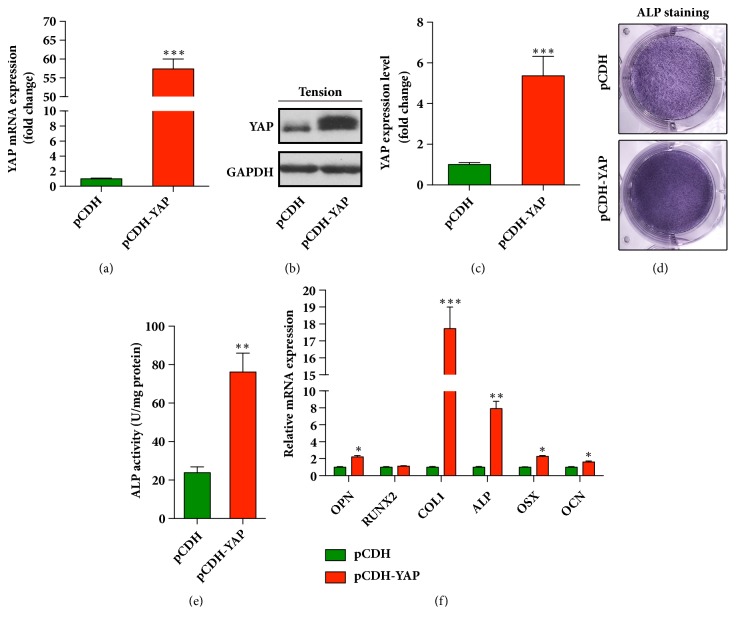
**Forced overexpression of YAP promotes the osteogenic differentiation of HPDLCs**. (a) YAP was overexpressed in HPDLCs using pCDH-YAP, and the mRNA expression level of YAP was detected by qRT-PCR. *∗∗∗*,* p* < 0.001. (b) The protein expression of YAP was analyzed using immunoblots. (c) Quantitative analysis of the blots (in (b)), the data was normalized to GAPDH. *∗∗∗*,* p* < 0.001. (d) Alkaline phosphatase activity of HPDLCs was enhanced by the overexpression of YAP. The positive cells for the ALP were stained with NBT-formazan (dark blue). (e) The ALP activities were quantified using an ALP reagent kit. The activities were normalized to the protein concentration. *∗∗*,* P* < 0.01. (f) Osteogenesis related genes were increased in the YAP overexpressed HPDLCs. The relative expression levels of osteopontin (OPN), RUNX2, collagen type 1 (COL1), alkaline phosphatase (ALP), osterix (OSX), and osteocalcin (OCN) were normalized to GAPDH. *∗*,* p* < 0.05; *∗∗*,* p* < 0.01; *∗∗∗*,* p* < 0.001. All data was based on three independent experiments.

On the other hand, we wanted to clarify whether overexpression of YAP could enhance the osteogenic differentiation of HPDLCs. Therefore, a lentiviral plasmid pCDH-YAP was constructed to forcedly overexpress YAP in HPDLCs. The non-cytotoxic effect of pCDH-YAP has been confirmed via trypan blue exclusion assay (Supplementary Figure 3). As shown in Figures 4(a)–4(c), the mRNA and protein expression level of YAP in HPDLCs were measured through real-time qPCR and western blots. The results demonstrated that the expression of YAP was significantly increased in the cells transfected with pCDH-YAP compared with that in control cells. The YAP-overexpressed HPDLCs were stretched in osteoblastic differentiation media for 3 days and then were explored for their potential of osteoblastic differentiation. Our results revealed that in the YAP overexpressing HPDLCs, ALP activity was increased significantly (Figures 4(d)-4(e)). Furthermore, the mRNA expression levels of osteogenesis-related genes, including OPN, collagen type 1, ALP, OSX and OCN, were measured. All of them were increased in the YAP overexpressing cells rather than the cells in the control group (Figure 4(f)). Taken together, these results suggested that forced up-regulation of YAP in HPDLCs enhanced their osteogenic differentiation potential.

### 3.5. Cytoskeletal Remodeling Is Necessary for the Activation of YAP and Cyclic Stretch-Induced Osteogenesis in HPDLCs

**Figure 5:**
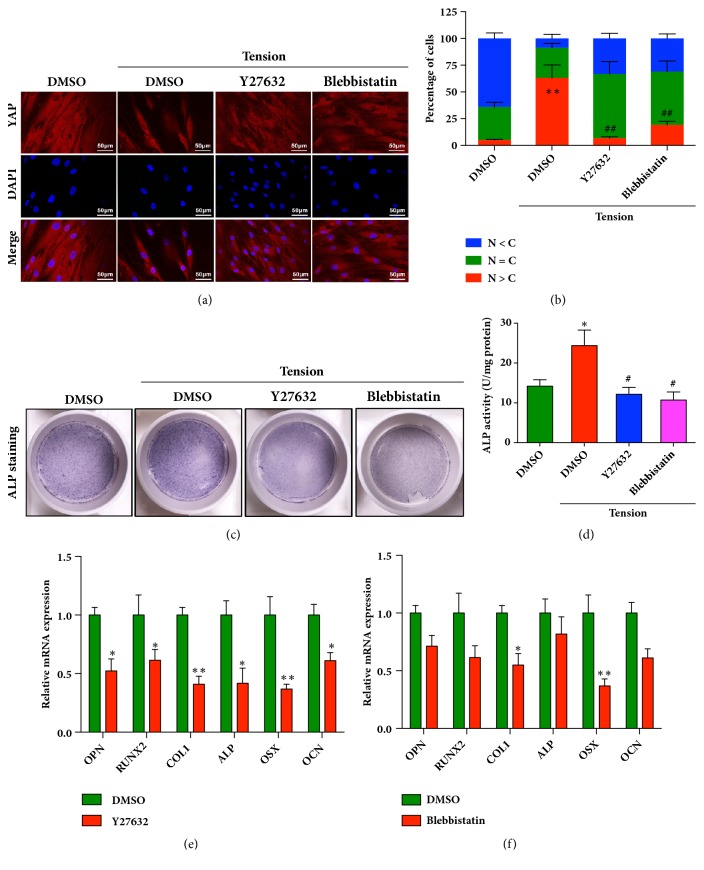
**Activation of YAP in HPDLCs was mediated by cytoskeleton remodeling**. (a) Y27632 (20 *μ*M) and Blebbistatin (20 *μ*M) suppressed the nuclear translocation of YAP in HPDLCs after cyclic stretching. Scale bars indicate 50 *μ*m. (b) Quantitatively analysis of the distribution patterns of YAP (in (a)). The patterns were categorized as mainly nuclear (N>C), diffuse (N=C), and mainly cytoplasmic (N<C). (c) Alkaline phosphatase activity was decreased in the Y27632- and Blebbistatin-treated HPDLCs after cyclic stretching. The positive cells were stained with NBT-formazan (dark blue). (d) The ALP activities were quantified using an ALP reagent kit. The activities were normalized to the protein concentration. *∗*,* P* < 0.05 vs control group; ^#^,* p* < 0.05 vs tension group. (e and f) The mRNA expression levels of osteogenesis related genes in HPDLCs were decreased in the presence of Y27632 (e) or Blebbistatin (f) with cyclic stretch treatment. The relative expression levels of YAP, osteopontin (OPN), RUNX2, collagen type 1 (COL1), and alkaline phosphatase (ALP) were normalized to GAPDH. *∗*,* p* < 0.05; *∗∗*,* p* < 0.01. All data was based on three independent experiments.

Mechanical stimulation, especially the stretch, could induce the cytoskeletal remodeling by activating Rho/ROCK/non-muscle myosin II (NMMII) signaling pathway, which further enhanced the nuclear translocation of YAP [29]. Therefore, we explored whether the cycling stretch could induce the cytoskeleton remodeling in HPDLCs and whether the increased cytoskeletal tension was crucial for the activation of YAP and the following enhanced osteoblastic differentiation. Thus, Y-27632 (20 *μ*M), the ROCK specific inhibitor, and blebbistatin (20 *μ*M), and the nonmuscle myosin ATPase inhibitor were used. As shown in Figures 5(a) and 5(b), cyclic stretch-induced nuclear localization of YAP was significantly abrogated in the presence of Y27632 and blebbistatin, suggesting that cytoskeletal tension was important for the activation of YAP in tension treated HPDLCs. Then, in the presence of Y27632 and blebbistatin, the HPDLCs were cultured in osteoblastic differentiation media and treated with the cyclic stretch. As shown in Figures 5(c) and 5(d), the alkaline phosphatase activities of the HPDLCs pretreated with Y27632 or blebbistatin were significantly reduced, indicating that the osteogenic differentiation potential of HPDLCs under cyclic tension force was impaired due to the blockage of cytoskeleton remodeling. Moreover, the expression levels of osteogenesis-related genes, including OPN, RUNX2, collagen type 1, and ALP, were also decreased in the HPDLCs that were pretreated with Y27632 or blebbistatin after cyclic stretch (Figures 5(e) and 5(f)). Collectively, these results suggested that the increased cytoskeletal remodeling played an important role in the cyclic stretch-induced osteogenesis by regulating YAP activity.

### 3.6. YAP Was Nuclear Translocated during the Orthodontic Tooth Movement in the Murine Model

**Figure 6:**
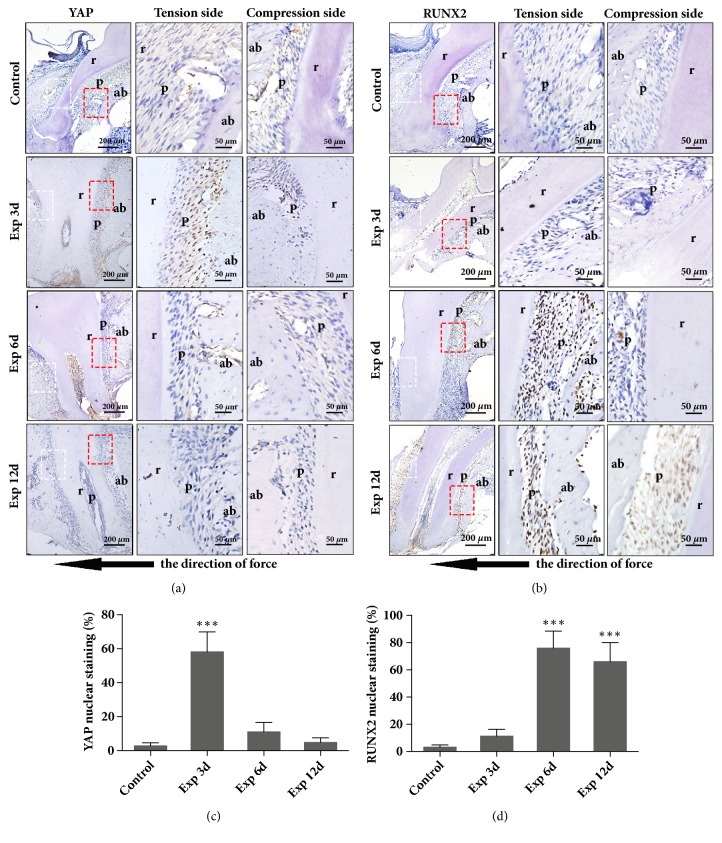
**Activation of YAP in the PDLCs in the OTM murine model**. Histological section at the tension side of the periodontium zone around the mesial root of right upper first molar during OTM was analyzed by immunohistochemical staining for YAP (a, brown) and RUNX2 (b, brown) in control group (Control) and experiment group after mechanical loading for 3 days (Exp 3d), 6 days (Exp 6d), and 12 days (Exp 12d). Nuclei were counterstained with hematoxylin (blue). The red boxed regions (tension side) and white boxed regions (compression side) were shown at a higher magnification. r for Root, ab for alveolar bone, and p for periodontium, while black arrow indicates the direction of force. The percentages of positive nuclear staining of YAP (c) and RUNX2 (d) at the tension side of periodontium were analyzed. *∗∗*, P < 0.01; *∗∗∗*, P < 0.01.

We further determined the YAP activation in periodontal ligaments during orthodontic tooth movement* in vivo*. Mice and rats were the most frequently used animal models in the research for the mechanical stimulation of alveolar bone formation during orthodontic tooth movement. To achieve molar movement, several different methods had been used, including inserting a compressed elastic band between upper first and second molar [30], or exerting orthodontic strains by an elastic ring or a coil spring [31, 32]. In our study, we bonded the upper first incisors to upper first molar with a coil spring in our mouse model to mimic orthodontic tooth movement as we previously reported [18]. RUNX2, which was reported to control the differentiation of osteoblast and bone formation [33], was also measured in the models. As shown in Figure 6(a), the positive staining of YAP was noticed in the nucleus of the PDLCs that were adjacent to alveolar bone on day 3 after mechanical loading, and the expression of YAP was then downregulated gradually from day 6 to day 12. Meanwhile, we noticed that the nuclear expression of RUNX2 was found in PDLCs from the day 3, which was gradually increased on day 6 and lasted until the day 12 (Figure 6(b)). The quantification of YAP and RUNX2 staining in the tension side has been showed in Figures 6(c) and 6(d). In addition, we noticed that the positive staining of YAP and RUNX2 was rarely observed in the compression side (Figures 6(a) and 6(b)). Considering that YAP regulated the osteoblastic differentiation of HPDLCs in vitro, we believed that the orthodontic force induced activation of YAP, further promoting the osteogenesis during OTM.

## 4. Conclusion

Orthodontic tooth movement is featured by bone deposition on the tension side and bone resorption on the compression side. During the process, the periodontal ligament, which is mechanosensitive and functionally heterogeneous, containing a subpopulation of cells capable of perceiving mechanical signals and converting them into biochemical signals, contributed to the bone formation on the tension side [3, 34]. Our research revealed that YAP served as an important regulator mediating the osteogenic differentiation of HPDLCs under cyclic stretch induced mechanical stimulations. The stimulation activated YAP signaling pathways through cytoskeleton remodeling. The highly expressed YAP might interact with RUNX2 and enhance its transcriptional activity during the osteogenesis. The results in the present study help us acquire further insights into the molecular mechanism underlying the osteogenic differentiation of HPDLCs during orthodontic tooth movement, which may hopefully provide a new therapeutic target for the rapid and efficient orthodontic treatment.

## Data Availability

The data used to support the findings of this study are available from the corresponding author upon request.

## Abbreviations

YAP: Yes-associated protein
OCN: Osteocalcin
OPN: Osteopontin
RUNX2: Runt-related transcription factor 2
COL1: Collagen type 1
ALP: Alkaline phosphatase
OSX: Osterix
PDLCs: Periodontal ligament cells
OTM: Orthodontic tooth movement.

## Supplementary Materials

Supplementary Materials Table  S1: Age and gender information of HPDLCs sample donor; Figure  S1: TAZ expression in human periodontal ligament cells (HPDLCs) under cyclic stretching. Figure  S2: YAP siRNA transfection shows no cytotoxic effect on the PDLCs. Figure  S3: YAP retroviral transfection shows no cytotoxic effect on the PDLCs.
