# Supplementary material for: Cyclic Stretch Enhances Osteogenic Differentiation of Human Periodontal Ligament Cells via YAP Activation
Source: Biomed Res Int. 2018 Nov 5;2018:2174824. doi: 10.1155/2018/2174824 (PMC6241358; doi:10.1155/2018/2174824)
Supplement: Supplementary Materials — Table S1: Age and gender information of HPDLCs sample donor; Figure S1: TAZ expression in human periodontal ligament cells (HPDLCs) under cyclic stretching. Figure S2: YAP siRNA transfection shows no cytotoxic effect on the PDLCs. Figure S3: YAP retroviral transfection shows no cytotoxic effect on the PDLCs. [file 2174824.f1.docx]

**Supplementary data for**

**Cyclic stretch enhances osteogenic differentiation of human periodontal ligament cells via YAP activation**

Yang Yang^1,2^, Bei-Ke Wang^1,2^, Mao-Lin Chang^1,2^, Zi-Qiu Wan^1,2^, Guang-Li Han^1,2^

^1^ State Key Laboratory Breeding Base of Basic Science of Stomatology (Hubei-MOST) and Key Laboratory for Oral Biomedicine of Ministry of Education (KLOBM), School and Hospital of Stomatology, Wuhan University, 237 Luoyu Road, Wuhan 430079, China; ^2^ Department of Orthodontics, School & Hospital of Stomatology, Wuhan University, Wuhan, China.

**Author contribution** Yang Yang and Bei-Ke Wang contributed equally to this work. All authors read and approved the final manuscript.

**Correspondence to** Guang-Li Han guanglihan@whu.edu.cn

**Table S1. Age and Gender information of HPDLCs sample donor**

| **Patient No.** | **#1** | **#2** | **#3** | **#4** | **#5** | **#6** | **#7** | **#8** | **#9** | **#10** |
| --- | --- | --- | --- | --- | --- | --- | --- | --- | --- | --- |
| **Age** | 17 | 21 | 16 | 22 | 14 | 18 | 18 | 24 | 20 | 12 |
| **Gender** | Male | Male | Female | Male | Male | Female | Male | Female | Male | Female |


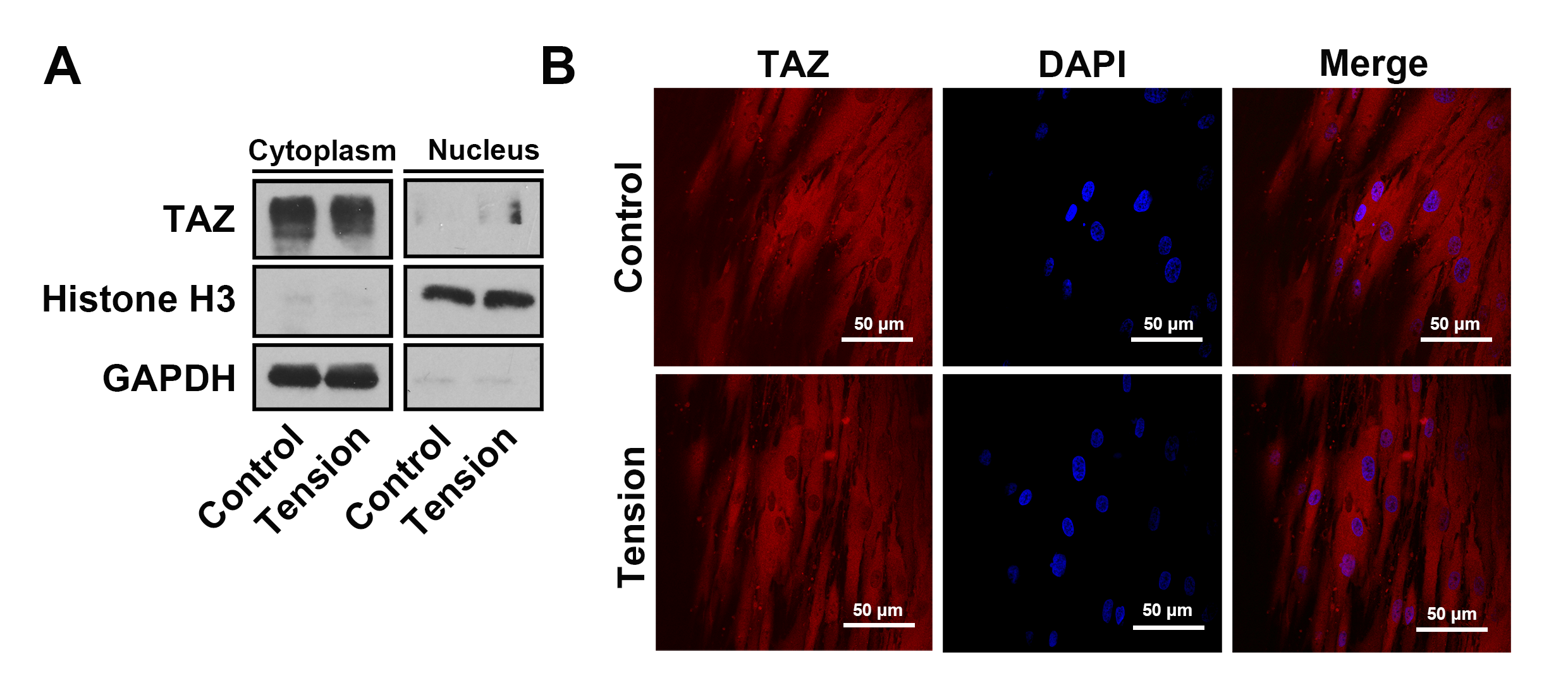


**Figure S1. TAZ expression in human periodontal ligament cells (HPDLCs) under cyclic stretching.** (A) Nuclear and cytoplasmic expression of TAZ in stretched HPDLCs. (B) Immunofluorescence of TAZ in cyclic stretched HPDLCs.

**Figure S2. YAP siRNA transfection shows no cytotoxic effects on the PDLCs.** The cell viability of the PDLCs has been detected via trypan assay after treated with siNC and siYAP for 48h. All the data was based on the three independent experiments.

**Figure S3. YAP retroviral transfection shows no cytotoxic effects on the PDLCs.** The cell viability of the PDLCs has been detected via trypan assay after treated with pCDH and pCDH-YAP for 48h. All the data was based on the three independent experiments.
